# Molecular dynamics simulations and docking of non-nucleoside reverse transcriptase inhibitors (NNRTIs): a possible approach to personalized HIV treatment

**DOI:** 10.1186/1758-2946-4-S1-P32

**Published:** 2012-05-01

**Authors:** Florian D Roessler, Oliver Korb, Andreas Bender, Werner Maentele, Peter J Bond

**Affiliations:** 1 Unilever Centre for Molecular Science Informatics, Department of Chemistry, University of Cambridge, Lensfield Road, Cambridge,CB2 1EW, UK; 2 The Cambridge Crystallographic Data Centre, 12 Union Road, Cambridge, CB2 1EZ, UK; 3 Institute of Biophysics, Johann Wolfgang Goethe-University Frankfurt, Max von Laue-Strasse 1, 60438 Frankfurt am Main, Germany

## 

The human immunodeficiency virus (HIV) is currently ranked sixth in the worldwide causes of death [1]. One treatment approach is to inhibit reverse transcriptase (RT), an enzyme essential for reverse transcription of viral RNA into DNA before integration into the host genome [2]. By using non-nucleoside RT inhibitors (NNRTIs) [3], which target an allosteric binding site, major side effects can be evaded. Unfortunately, high genetic variability of HIV in combination with selection pressure introduced by drug treatment enables the virus to develop resistance against this drug class by developing point mutations. This situation necessitates treatment with alternative NNRTIs that target the particular RT mutants encountered in a patient.

Previously, proteochemometric approaches have demonstrated some success in predicting binding of particular NNRTIs to individual mutants; however a structurebased approach may help to further improve the predictive success of such models. Hence, our aim is to rationalize the experimental activity of known NNRTIs against a variety of RT mutants by combining molecular modeling, long-timescale atomistic molecular dynamics (MD) simulation sampling and ensemble docking. Initial control experiments on known inhibitor-RT mutant complexes using this protocol were successful, and the predictivity for further complexes is currently being evaluated. In addition to predictive power, MD simulations of multiple RT mutants are providing fundamental insight into the dynamics of the allosteric NNRTI binding site which is useful for the design of future inhibitors. Overall, work of this type is hoped to contribute to the development of predictive efficacy models for individual patients, and hence towards personalized HIV treatment options.
